# Comparison of the effects of patient controlled analgesia (PCA) using dexmedetomidine and propofol during septoplasty operations: a randomized clinical trial

**DOI:** 10.1186/s40064-016-2245-y

**Published:** 2016-05-10

**Authors:** Başak Akça, Ayhan Arslan, Aysun Ankay Yılbaş, Özgür Canbay, Nalan Çelebi

**Affiliations:** Department of Anesthesiology, Faculty of Medicine, Hacettepe University, 06100 Sıhhiye, Ankara, Turkey; Department of Anesthesiology, Fatsa State Hospital, Ordu, Turkey

## Abstract

**Introduction:**

Septoplastical surgery to correct septum deviation can be performed under either local or general anesthesia. During local anesthesia, sedation helps to provide minimum anxiety/discomfort. Our aim was to evaluate the effects of patient-controlled analgesia using dexmedetomidine and propofol on sedation level, analgesic requirement, and patient satisfaction.

**Study design:**

A prospective, randomized-parallel clinical study.

**Methods:**

Fifty patients undergoing septoplastical surgery at our university hospital were randomized into two groups. A nasopharyngeal cotton tampon soaked in 0.25 % adrenaline solution was placed, and 1 mg midazolam and 1 mcg/kg fentanyl were applied 5 min before the injections of a surgical local anesthetic. Loading dose was 0.5 mg/kg propofol (Group I) and 1 mcg/kg dexmedetomidine (Group II). The sedation was sustained by a bolus dose of 0.2 mg/kg and continuous basal infusion dose of 0.5 mg/kg/h propofol in Group I, or by a bolus dose of 0.05 µg/kg and continuous basal infusion dose of 0.4 mcg/kg/h dexmedetomidine in Group II. The primary outcomes were patient satisfaction via patient-controlled anesthesia and analgesic demand. Secondary outcomes were sedation level of patients under local anesthesia.

**Results:**

In Group II, SpO_2_ levels were significantly higher than in Group I. Intraoperative and postoperative analgesic requirements were lower in Group II than in Group I. There were no statistically significant differences in patient satisfaction, hemodynamic parameters, nausea and vomiting between the two groups.

**Conclusion:**

Dexmedetomidine can be used safely as an analgesic and sedation drug in septoplastic surgery.

## Background

Septoplastical surgery to correct septum deviation can be performed under either local or general anesthesia. General anesthesia provides a safe airway throughout the surgery, a comfortable surgical procedure and eliminates the need for patient cooperation. On the other hand, local anesthesia has advantages of not requiring endotracheal intubation or mechanical ventilation. Although local anesthesia has less postoperative side effects and is less invasive than general anesthesia, it is not a process that is free of complications (Ridenour 1998). The purpose of using sedation during local anesthesia is to provide minimum anxiety/discomfort for patients.

During the surgery, sedation requirements change frequently depending on the stages of the operation. Intravenous sedative medications allow dosage adjustments in conscious patients. (Alhashemi and Kaki 2006) compared patient-controlled and anesthesiologist-controlled sedation, and found that during the latter, more medication was used, sedation was deeper and recovery time was longer. In addition, patient-controlled sedation allows ease of control of the depth of sedation and helps provide minimum anxiety/discomfort during surgery (Joo et al. 2001).

Nausea, vomiting and nasal bleeding are more frequent in patients undergoing general anesthesia than in patients undergoing local anesthesia. Sufficient bleeding and pain control of the nasal septum improves patient cooperation and patient handling and as a consequence; patient satisfaction at the end of the surgery (Fedok et al. 2000). Two commonly used drugs for patient-controlled sedation are dexmedetomidine and propofol. Dexmedetomidine is an α2 adrenergic receptor (adrenoceptor) agonist which is used for its unique properties for sedation and analgesia during the perioperative period. Because of its potent sedative, analgesic, perioperative sympatholytic effects, reduced anesthetic demand and cardiovascular stabilizing effects, dexmedetomidine became the center of interest for many recent studies. In addition, it does not cause respiratory depression. Dexmedetomidine activates the receptors in the brain and spinal cord and causes hypotension, bradycardia, sedation and analgesia (Gertler et al. 2001 Jan). On the other hand, propofol is a short-acting anesthetic with short half-life, controllable dose–effect power and stable elimination rate. Propofol is sedative and anxiolytic in low doses but hypnotic in high doses. It has low to no amnestic effects. Depending on the dosage and the injection rate, a 15–25 % decrease in systolic, diastolic and mean arterial pressure (MAP) occurs in healthy patients, which makes it a widely preferred agent in septoplastic surgery. Nevertheless, propofol has no analgesic effect (Rajapakse et al. 2003; Köksal 2007; Janzen et al. 1999).

In this study, our aim was to compare the sedation level and analgesic demand of patient-controlled analgesia (PCA) using dexmedetomidine and propofol.

## Methods

### Trial design

After Hacettepe University Medical Research Ethics Committee approval and informed consent, 50 patients aged 18–50 years who were undergoing septoplastical surgery were included in this prospective, randomized, parallel-group clinical study. The study was conducted at Hacettepe University Hospital. A single surgeon operated all patients. Patients with advanced heart, lung and kidney diseases; heavy bronchial asthma; cardiac block; long-term opioid, sedative and β-blocker usage; obesity with body mass index (BMI) >35 kg/m^2^; and a history of allergic reaction to medications used in this study were excluded from the study.

### Randomization

The 50 patients were randomly assigned to two groups (allocation ratio 1:1). Randomization was done by the envelope draw method. Patients were informed about the usage of PCA before the surgery. Standard monitorization (continuous electrocardiogram, non-invasive blood pressure monitoring, pulse oximeter, capnography) was applied. Th vital signs of all patients were recorded every 5 min.

### Intervention

A nasopharyngeal cotton tampon soaked in a solution of 0.25 % adrenaline (Adrenalin^®^; Biofarma, Turkey) was placed. During the procedure, 3–5 L/min O_2_ was provided to the patients via an oral cannula. Five minutes before the injection of a surgical local anesthetic, 1 mg midazolam (Dormicum^®^; Roche, Turkey) and 1 mcg/kg fentanyl (Fentanyl Citrate^®^; Abbott, North Chicago, USA) were applied intravenously.

#### **Group I**

1 mg midazolam and 1 mcg/kg fentanyl were applied 5 min before the application of surgical local anesthetic injection. After the delivery of a loading dose of 0.5 mg/kg propofol in 10 min (Propofol-Lipuro^®^ 1 %; Braun, Melsungen, Germany), local anesthesia with 0.5 % prilocaine hydrochloride–epinephrine solution (Jetokain^®^; Adeka, Turkey) was applied by the surgeon. The sedation was sustained by a bolus dose of 0.2 mg/kg and a continuous basal infusion dose of 0.5 mg/kg/h propofol.

#### **Group II**

1 mg midazolam and 1 mcg/kg fentanyl were applied 5 min before the surgical local anesthetic injection. After the delivery of a loading dose of 1 mcg/kg dexmedetomidine in 10 min (Precedex^®^; Hospira, Lake Forest, USA), local anesthesia with 0.5 % prilocaine hydrochloride–epinephrine solution (Jetokain^®^) was applied by the surgeon. The sedation was sustained by a bolus dose of 0.05 µg/kg and a continuous basal infusion dose of 0.4 mcg/kg/h dexmedetomidine.

When sufficient analgesia could not been achieved [visual analogue score (VAS) >4)], 1 mcg/kg fentanyl was added to both groups and the dose was recorded. In addition, nausea and vomiting were evaluated at the end of each operation.

### Outcomes

The primary outcomes were patient satisfaction and analgesic demand. Patient satisfaction was evaluated using two 4-point scales (1: bad; 4: very good): the visual analogue scale (VAS) and the verbal rating scale (VRS) postoperatively. Secondary outcomes were sedation level evaluated by the observer’s assessment of alertness/sedation (OAA/S) perioperatively.

### Statistical analysis

All statistical analyses were performed with SPSS v. 22.0. Shapiro–Wilk test was used as normality test. Continuous variables were compared using Student’s *t-*test and Mann–Whitney *U*-test when the data were not normally distributed. Categorical variables were compared using Pearson’s Chi squared test and Fisher’s exact test. For responses at different time points, percent changes were calculated according to baseline measurement. These percent changes were compared using Mann–Whitney *U*-test for two groups. A p-value <0.05 was considered statistically significant. A sample size of 28 patients (14 in each group) was calculated as necessary to detect a 1.5 point difference in VAS scores (expected SD ± 1.5) with a power of 80 % and α error of 0.05. We decided to start with 25 patients in each group in case some patients could be excluded due to intraoperative complications or need for general anesthesia.

## Results

Of the 50 patients initially enrolled, 49 were finally included in our study. One patient who had been intubated after sedation protocol from the propofol group was not included. There was no statistically significant difference between the two groups in terms of BMI, gender or age (Table 1).

**Table 1:** Demographic data of the study patients

	Group I		Group II		p
	$\bar{x}$ (SD)	Median (range)	$\bar{x}$ (SD)	Median (range)	
Age	30.08 (5.92)	29.50 (22–43)	27.40 (9.22)	25 (18–47)	0.067
BMI	22.85 (2.52)	22.16 (18.97–29.06)	21.45 (2.58)	21.48 (17.30–26.42)	0.061

$\bar{x}$ Arithmetic average, *SD* standard deviation, *BMI* body mass index

When Groups I and II were compared in terms of MAP, a significantly greater decrease in Group I was observed at 10 min postoperatively (p < 0.05). In other time points, the difference was not statistically significant (Table 2).

**Table 2:** Mean arterial pressure in Group I and II

Measurement time	Group I	Group II	p
	Median (range)	Median (range)	
Preoperative	95 (69–117)	94 (73–109)	0.36
10 min	74 (44–111)	80 (72–103)	0.02*
20 min	77 (57–92)	78 (68–107)	0.38
30 min	80 (54–103)	82 (65–102)	0.11
40 min	77 (51–121)	83 (68–103)	0.07
50 min	74 (52–97)	80 (66–101)	0.05
60 min	76 (54–108)	82 (67–99)	0.05
70 min	74 (52–110)	79 (64–93)	0.68

* There is a significant decrease in MAP at 10 min

When Groups I and II were compared in terms of heart rate, a significantly greater decrease in Group I was observed at 20 min postoperatively (p < 0.05). In other time points, the difference was not statistically significant (Table 3).

**Table 3:** Heart rate in Groups I and II

Measurement time	Group I	Group II	p
	Median (range)	Median (range)	
Preoperative	86 (61–105)	78 (63–100)	0.08
10 min	80 (57–91)	83 (67–108)	0.42
20 min	74 (53–89)	80 (63–95)	0.02*
30 min	77 (63–87)	80 (66–88)	0.06
40 min	75 (54–90)	75 (65–91)	0.90
50 min	76 (53–92)	76 (62–83)	0.56
60 min	78 (53–90)	78 (60–87)	0.49
70 min	80 (62–88)	75 (55–86)	0.06

* Statistically significant

When Groups I and II were compared in terms of SpO_2_, a significantly greater decrease in Group I was observed at 30, 40, 50, 60 and 70 min postoperatively (p < 0.05). In other time points, the difference was not statistically significant (Table 4).

**Table 4:** SpO_2_ in Groups I and II

Measurement time	Group I	Group II	p
	Median (range)	Median (range)	
Preoperative	99 (95–100)	98 (97–99)	0.08
10 min	98 (92–100)	98 (96–99)	0.99
20 min	98 (94–100)	99 (97–100)	0.15
30 min	96 (93–100)	98 (96–100)	<0.01*
40 min	95 (93–99)	98 (96–100)	<0.01*
50 min	95 (94–98)	99 (96–100)	<0.01*
60 min	97 (92–100)	99 (97–100)	<0.01*
70 min	98 (95–99)	99 (97–100)	0.01*

* Statistically significant

When Groups I and II were compared in terms of VRS scores, a significantly greater decrease in Group I was observed at 10, 30, 50, 60 and 70 min postoperatively (p < 0.05). At 40 min postoperatively, the difference was not statistically significant (Table 5).

**Table 5:** Verbal rating scale scores in Groups I and II

Measurement time	Group I	Group II	p
	Median (range)	Median (range)	
10 min	0.0 (0.0–3.0)	0.0 (0.0–1.0)	<0.01*
20 min	1.0 (0.0–3.0)	0.0 (0.0–2.0)	<0.01*
30 min	0.5 (0.0–3.0)	0.0 (0.0–2.0)	0.01*
40 min	0.0 (0.0–3.0)	0.0 (0.0–2.0)	0.59
50 min	0.5 (0.0–2.0)	0.0 (0.0–2.0)	<0.01*
60 min	1.0 (0.0–3.0)	0.0 (0.0–2.0)	<0.01*
70 min	0.0 (0.0–4.0)	0.0 (0.0–2.0)	0.51

* Statistically significant

When Groups I and II were compared in terms of OAA/S scores, a significantly greater decrease in Group I was observed at 20, 30, 40, 60 and 70 min postoperatively (p < 0.05). In other time points, the difference was not statistically significant (Table 6).

**Table 6:** Observer’s assessment of alertness/sedation (OAA/S) scale scores in Groups I and II

Measurement time	Group I	Group II	p
	Median (range)	Median (range)	
10 min	5 (3–5)	5 (3–5)	0.69
20 min	5 (3–5)	5 (3–5)	0.03*
30 min	5 (2–5)	5 (5–5)	<0.01*
40 min	5 (3–5)	5 (5–5)	<0.01*
50 min	5 (3–5)	5 (4–5)	0.17
60 min	5 (3–5)	5 (5–5)	<0.01*
70 min	5 (4–5)	5 (5–5)	0.03*

* Statistically significant

Compared to Group I, Group II showed a significantly greater decrease in VAS scores (p < 0.05) at 30 min postoperatively. In other time points, the difference was not statistically significant (Table 7).

**Table 7:** Visual analogue scale scores in Groups I and II

Measurement time	Group I	Group II	p
	Median (range)	Median (range)	
30 min	5 (1–7)	4 (1–6)	0.01*
60 min	4 (0–8)	3 (0–5)	0.12
120 min	1 (0–7)	2 (0–4)	0.98

* Statistically significant

When Groups I and II were compared in terms of total fentanyl dosage, Group I required significantly more doses than Group II (p < 0.05) (Table 8).

**Table 8:** Total fentanyl dosage in Groups I and II

	Group I	Group II	p
	Median (range)	Median (range)	
Total	225 (150–300)	45 (25–50)	<0.01*

* Statistically significant

No statistically meaningful difference was observed in postoperative nausea and vomiting between the two groups (Tables 9, 10).

**Table 9:** Frequency of postoperative nausea in Groups I and II

Measurement time	Group I (%)	Group II (%)	p
30 min	8.3	20.0	0.417
60 min	12.5	20.0	0.702
120 min	12.5	8.0	0.667

**Table 10:** Frequency of postoperative vomiting in Groups I and II

Measurement time	Group I (%)	Group II (%)	p
30 min	0	4	1.000
60 min	8.3	4	0.609
120 min	8.3	4	0.609

## Discussion

In this study, we compared dexmedetomidine and propofol in terms of sedation, analgesic demand, and patient satisfaction via PCA in 50 patients who underwent septoplastic surgery. We observed that dexmedetomidine provided enough sedation and analgesic levels without causing respiratory depression. We also found that compared to propofol, dexmedetomidine provided stable hemodynamics and satisfactory sedation while reducing the need for additional anesthesia by decreasing the requirement for intraoperative fentanyl. No statistically significant difference was observed in postoperative patient satisfaction.

When it is applied by experienced surgeons, septoplasty is mostly a daily operation owing to the short duration of surgery and easy postoperative care (Ridenour 1998). Because of the innervational characteristics of the septum, a good intraoperative hemorrhage control and an effective postoperative analgesia requirement is highly recommended (Alhashemi and Kaki 2006).

Discussions are still continuing whether septoplasty should be applied under general anesthesia or local anesthesia with sedation. Obtaining surgical analgesia and amnesia without requiring an airway instrumentation and with short hospital stays are the biggest advantages of local anesthesia with sedation (Joo et al. 2001). In a study by Fedok and colleagues that included 177 nasal septoplasty and endoscopic sinus surgery patients, total surgery time, recovery unit and discharge from the hospital were shorter in the local anesthesia with sedation group than in the general anesthesia group Fedok et al. (2000). In addition, the frequency of postoperative nausea and epistaxis was higher in the general anesthesia group (Fedok et al. 2000). In another study including 197 nasal fracture patients, subjective patient assessments were significantly better in the local anesthesia with sedation group; 69 % of those patients stated that they would accept to be operated on a second time using the same method (Rajapakse et al. 2003).

Many different drugs (e.g., propofol, benzodiazepines and opioids) are used to provide sedation in septoplasty. Among them, propofol is associated with complications such as delayed surgery or hospitalization, especially in elderly patients (Köksal 2007; Janzen et al. 1999; Weinbroum et al. 2001; Wong and Merrick 1996). Myoclonus, muscle twitch and hiccups are also among the known side effects of propofol use. In our study, although myoclonus, muscle twitch, and hiccups were not observed, 41.6 % of the patients stated having pain on the back of the hand at the site of propofol injection. A patient in the propofol group required intubation because of apnea development and insufficient anesthesia. Additionally, oxygen saturation values were lower in this group than the dexmedetomidine group, but these values were not statistically significant Thus, patients using propofol need to be monitored closely, and additional analgesic medication is often required even during PCA (Salmon et al. 1992; Sebel and Lowdon 1989; Peacock et al. 1990; Grounds et al. 1987; Duke 2006). In our study, unlike propofol, dexmedetomidine promoted the desired sedation levels. No respiratory depression was observed when additional analgesic (an opioid) was required.

Uzumcugil and colleagues compared propofol–fentanyl and dexmedetomidine infusion for laryngeal mask airway insertion and found that dexmedetomidine provided more stable hemodynamics in geriatric patients (Uzümcügil et al. 2008). In our study, postoperative MAP values were significantly lower than preoperative values in both groups. Clinically, the decrease in MAP was not important and it was within the desired levels for hypotensive anesthesia, which is required for septoplastic surgeries. In the propofol group, the heart rate at 20 min was lower than in the dexmedetomidine group. These low values were statistically significant, yet were not clinically within bradycardia border limits, thus not requiring any intervention.

In our study, at 30, 40, 50, 60 and 70 min postoperatively, the SpO2 values were lower in propofol group than in the dexmedetomidine group. The decrease at these saturation levels was not deep enough to pose any clinical risk. In other words, no clinically desaturation developed in either group. Only one patient in the propofol group required intubation due to apnea, and was excluded from the study right after intubation. In another study performed by Kenan and his colleagues, comparing dexmedetomidine and propofol in ESWL, SpO_2_ values were significantly higher (Kaygusuz et al. 2008). In the same study, although dexmedetomidine lowered the respiratory rate compared to propofol, SpO_2_ values in the dexmedetomidine group were higher. The authors attributed these results to propofol causing minimal decrease in respiratory rate and tidal volume at sedative dosage. We suggest that the higher oxygen saturation values in the dexmedetomidine group might be associated to respiratory depression effect of propofol as well as high fentanyl requirement for analgesia (Kaygusuz et al. 2008; Hall et al. 2000).

Turgut and colleagues significantly higher postoperative additional analgesic requirement for the fentanyl group than for the dexmedetomidine group (Turgut et al. 2008). In another study by Kenan and colleagues in which sedation with dexmedetomidine and propofol was compared in ESWL, VAS values in the dexmedetomidine group at 25 and 35 min were significantly lower than in the propofol group. Dexmedetomidine was found to be better than propofol for providing sedation and analgesia (Kaygusuz et al. 2008). In our study, when intraoperative VRS, postoperative VAS values and analgesic effectiveness were compared, statistically significant lower values were observed in the dexmedetomidine group than in the propofol group at 10, 20, 30, 50, 60 and 70 min. Postoperative VAS at 30 min was significantly lower in the dexmedetomidine group than in the propofol group. The results are similar to those found in the literature.

Turgut and his colleagues showed that nausea and vomiting were statistically higher in the fentanyl group (Turgut et al. 2008). In the study conducted by Koroglu and colleagues, propofol and dexmedetomidine were compared in MRI of children and neither nausea nor vomiting was reported (Koroglu et al. 2006). Kenan and colleagues compared dexmedetomidine and propofol in ESWL procedure. 2 patients in dexmedetomidine and 5 patients in propofol group reported having nausea, only 2 patients in propofol group vomited, yet with no statistically significant difference (Kaygusuz et al. 2008). In our study, in accordance to other studies, no statistically significant difference was found between propofol and dexmedetomidine groups in nausea and vomiting incidence. We attributed this result to the antiemetic properties of propofol at sub-hypnotic doses (Turgut et al. 2008), although more fentanyl was applied.

The sedation level required to tolerate the operation by the patients may be different than the sedation level provided by the anesthesiologist. During the surgery, sedation requirements change constantly.

## Conclusion

In our study dexmedetomidine provided adequate intraoperative analgesia as well as satisfactory sedation without suppressing respiration. In terms of postoperative patient satisfaction, both dexmedetomidine and propofol were similar. Therefore, dexmedetomidine can be safely used as a sedative agent via PCA in patients having surgery under local anesthesia with sedation.
